# Attention Network Dysfunction in Bulimia Nervosa - An fMRI Study

**DOI:** 10.1371/journal.pone.0161329

**Published:** 2016-09-08

**Authors:** Jochen Seitz, Manuel Hueck, Brigitte Dahmen, Martin Schulte-Rüther, Tanja Legenbauer, Beate Herpertz-Dahlmann, Kerstin Konrad

**Affiliations:** 1 Department of Child and Adolescent Psychiatry, Psychotherapy and Psychosomatics, University Hospital, RWTH University Aachen, Aachen, Germany; 2 Department of Cognitive Neuroscience, Faculty of Psychology and Cognitive Neuroscience, Maastricht University, Maastricht, The Netherlands; 3 JARA-BRAIN, 52074 Aachen, Germany; 4 LWL Child and Adolescent Psychiatry, University Hospital Hamm, Ruhr-University Bochum, Hamm, Germany; 5 Psychiatric Outpatient Clinic, University Mainz, Mainz, Germany; Chinese Academy of Sciences, CHINA

## Abstract

**Objective:**

Recent evidence has suggested an increased rate of comorbid ADHD and subclinical attentional impairments in bulimia nervosa (BN) patients. However, little is known regarding the underlying neural mechanisms of attentional functions in BN.

**Method:**

Twenty BN patients and twenty age- and weight-matched healthy controls (HC) were investigated using a modified version of the Attention Network Task (ANT) in an fMRI study. This design enabled an investigation of the neural mechanisms associated with the three attention networks involved in alerting, reorienting and executive attention.

**Results:**

The BN patients showed hyperactivation in parieto-occipital regions and reduced deactivation of default-mode-network (DMN) areas during alerting compared with HCs. Posterior cingulate activation during alerting correlated with the severity of eating-disorder symptoms within the patient group. Conversely, BN patients showed hypoactivation during reorienting and executive attention in anterior cingulate regions, the temporo-parietal junction (TPJ) and parahippocampus compared with HCs, which was negatively associated with global ADHD symptoms and impulsivity, respectively.

**Discussion:**

Our findings demonstrate altered brain mechanisms in BN associated with all three attentional networks. Failure to deactivate the DMN and increased parieto-occipital activation required for alerting might be associated with a constant preoccupation with food or body image-related thoughts. Hypoactivation of executive control networks and TPJ might increase the likelihood of inattentive and impulsive behaviors and poor emotion regulation. Thus, dysfunction in the attentional network in BN goes beyond an altered executive attentional domain and needs to be considered in the diagnosis and treatment of BN.

## Introduction

Bulimia nervosa (BN) is a chronic and difficult-to-treat disease with typical onset in adolescence or young adulthood. BN involves recurrent episodes of binge eating followed by self-induced compensatory strategies, such as vomiting, to avoid weight gain [1,2]. Patients typically show body image distortion, impulsive behavior, a loss of self-control during binge episodes, often sensation-seeking and problems with emotion regulation [3].

Recently, it has been suggested that, in addition to behavioral impulsivity, attention problems occur in patients with BN [4–7] but see Galderisi et al. [8] for opposing findings. Although these attentional impairments are often subclinical [9], BN is also associated with increased rates of attention deficit hyperactivity disorder (ADHD) [5,10,11]. Moreover, ADHD in childhood is associated with more severe eating-disorder symptoms in BN patients in adulthood [5,12,13].

Some studies have provided neuropsychological evidence of deficits in executive or selective attention in BN patients compared with healthy controls (HCs) during Go/Nogo, Degraded Signal, Stop-signal and Simon and Stroop tasks [5]. During these tasks, patients with BN often perform worse than HC; however, deficits are often small and in the subclinical range (for a recent review, see [9]) and co-vary in part with depressive comorbidity [14]. In a behavioral study of 57 patients with BN versus 40 HC [5], we recently observed deficits in Go/NoGo and Incompatibility tasks that were in part independent of depressive symptoms, in particular slower reaction times and larger standard deviations. Interestingly, clinical inattention scores explained more variance in eating-disorder symptoms than did impulsivity, suggesting an important role of attention deficits in BN.

At the neural level, very few studies have investigated attentional networks in BN and focused solely on the executive control system with divergent results. Marsh et al. (2009, 2001) demonstrated fronto-striatal hypoactivation in patients with BN during conflict trials in the Simon Spatial Task [15,16]. In contrast, Lock and colleagues [17] reported hyperactivation in fronto-parietal brain regions including the anterior cingulate cortex (ACC) and the temporal cortex and hypothalamus in a mixed group of patients with binging and purging Anorexia Nervosa or BN during a Go/NoGo paradigm.

Focusing merely on executive attention may not be sufficient to provide a complete picture of attentional dysfunction in eating disorders and may contribute to overlooking more basic deficits in attentional functions. Such basic deficits may, however, play an important role for complex cognitive processes, such as executive attention and distractibility. Therefore, it is important to assess different attentional processes based on neuroscientifically grounded models of attention.

Posner and Petersen have developed an influential model of attentional functions [18,19]. In this model, independent neural networks are assumed to underlie various attentional functions, such as alerting, orienting/reorienting, and executive control. Alerting is defined as achieving and maintaining an alert state following a warning cue. Orienting and reorienting is required when stimuli occur outside the current focus of attention, and executive control is defined as resolving conflict among more than one possible response. The alerting system has been associated with right frontal and bilateral parietal regions [18,20], orienting and reorienting of attention is thought to be controlled by a network around the right temporo-parietal junction (TPJ) and the right inferior frontal gyrus. The executive attentional network is assumed to include the ACC and bilateral prefrontal cortex [18,20]. Based on this model, the attention network task has been developed [21] which has been used widely in the past to investigate the behavioral and neural mechanism of attentional dysfunction across a variety of psychiatric and neurological diseases and in participants at-risk. These studies provided evidence for specific behavioral deficits in the basic alerting system in participants with Major Depression [22], for orienting deficits in participants with Autism and for executive attentional control deficits in participants with ADHD or Schizophrenia (see [23] for a review). Neurally, the task has proven even more sensitive for between-group differences and has shown abnormal neural mechanism associated with distinct attentional networks in participants with ADHD [24] or Schizophrenia [25] with associations between specific neural network dysfunction and severity of psychopathological symptoms. However, so far, the task has not been used in eating disorder research. Thus, following the attentional deficits discovered on the behavioral level in patients with BN in our previous study [5], the aim of this current study was to investigate the neural differences in these three attentional networks in adolescents and young adults with BN compared to control participants using an event-related fMRI study. We used a modified version of the Attention Network Task (ANT) [20,24] to investigate the neural substrates of alerting, reorienting and executive attention. Furthermore, to investigate the association between dysfunctional attentional networks and clinical parameters, we correlated brain activation in these areas with symptoms of ADHD, impulsivity and eating disorders. We hypothesized hypoactivation in the brain-network regions associated with executive control, potentially reflecting the reduced ability of patients with BN to properly engage in conflict resolution and self-control. For alerting and reorienting of attention, we expected dysfunctional neural-activation patterns, potentially reflecting difficulties to properly focus attention on upcoming events and to adequately shift attention when needed respectively.

## Methods

### Participants

Twenty-one female adolescents and young adults with BN based on DSM-IV and twenty age- and body mass index (BMI)-matched female HC participated in this study. The BN participants were recruited consecutively by the in- and outpatient clinics of the Department of Child and Adolescent Psychiatry of Aachen University Hospital and the University Outpatient Clinic in Mainz, Germany, both specialist centers for eating disorders. Exclusion criteria were a history of psychosis, substance abuse and IQ<80. Individuals receiving medication were not excluded. The HC group was recruited via flyers and newspaper ads that did not refer to the aims of the study (for more information see S1 File).

Three patients improved partially between the time of inclusion into the study and MRI assessment. Similar to Marsh et al. [15], we chose to include them as BN symptoms typically vary over time [26] and this study is thus a good representation of the general population of BN adolescents and young adults. The BN group comprised 7 inpatients and 14 outpatients. In- and outpatients did not differ significantly in age, BMI or clinical parameters. One BN patient had to be excluded post-hoc from the study due to technical difficulties with data acquisition during scanning. The mean duration of illness was 3.1 years. Four patients met criteria for current major depression, two received a diagnosis of AD(H)D, and five received medication (serotonin reuptake inhibitors) during the study. All of the demographic and clinical characteristics are presented in Table 1 and S1–S2 Tables. Patients from Mainz performed the scanning session Aachen in order to avoid scanner hardware differences. This study was approved by the Ethics Committee of Aachen University Hospital. The participants and their legal guardians (if applicable), provided informed written consent before inclusion in the study.

Further details on the behavioral assessments, MRI assessment and processing are described in the supplemental data.

**Table 1:** Demographics and clinical characteristics of female adolescents with bulimia nervosa and healthy controls.

	Bulimia Nervosa (N = 20)		Healthy controls (N = 20)		Analysis		
	Mean	SD	Mean	SD	t	df	p
Age (years)	18.71	2.53	17.90	1.35	1.26	38	n.s.
IQ	101.95	7.73	102.00	10.20	-0.02	36	n.s.
Body Mass Index (kg/m²)	21.44	2.52	20.34	2.21	1.45	37	n.s.
SIAB-Ex total score	79.8	30.2					
Objective binge episodes (per week)	4.40	2.52					
Vomiting episodes (per week)	5.64	3.92					
WRI total score	21.39	13.89	6.40	4.68	-4.55	36	0.000
BIS impulsivity score	64.83	15.26	51.15	9.61	3.34	36	0.002
BDI-2 depression score	22.61	14.50	3.95	5.22	5.39	36	0.000
SCL-90 anxiety score	8.44	9.91	1.45	2.01	3.09	36	0.004
ANT reaction times all trials (msec)	633.70	59.34	659.41	96.10	0.90	38.00	n.s.
ANT standard deviation all trials (msec)	103.09	20.90	119.36	36.53	1.73	38.00	n.s.
ANT misses (%)	0.29	0.49	0.40	0.59	0.62	38.00	n.s.
ANT errors (%)	1.85	1.40	2.45	2.29	0.99	38.00	n.s.
ANT Alerting effect (msec)	56.70	38.80	41.36	33.24	-1.34	38.00	n.s.
ANT Reorienting effect (msec)	97.91	49.47	93.34	53.20	-0.28	38.00	n.s.
ANT Executive Control effect (msec)	88.04	26.04	91.65	33.90	0.38	38.00	n.s.

SD: Standard deviation; T: Student’s t-test T-Value; df: degrees of freedom; p: p-value, uncorrected; SIAB-Ex: Structured Interview for Anorexia and Bulimia; WRI: Wender-Reimherr Interview for ADHD; BIS: Barratt Impulsivity Scale; BDI: Beck’s Depression Inventory; SCL-90: Symptoms Check List, ANT: Attention Network Task.

### Task

**Fig 1:**
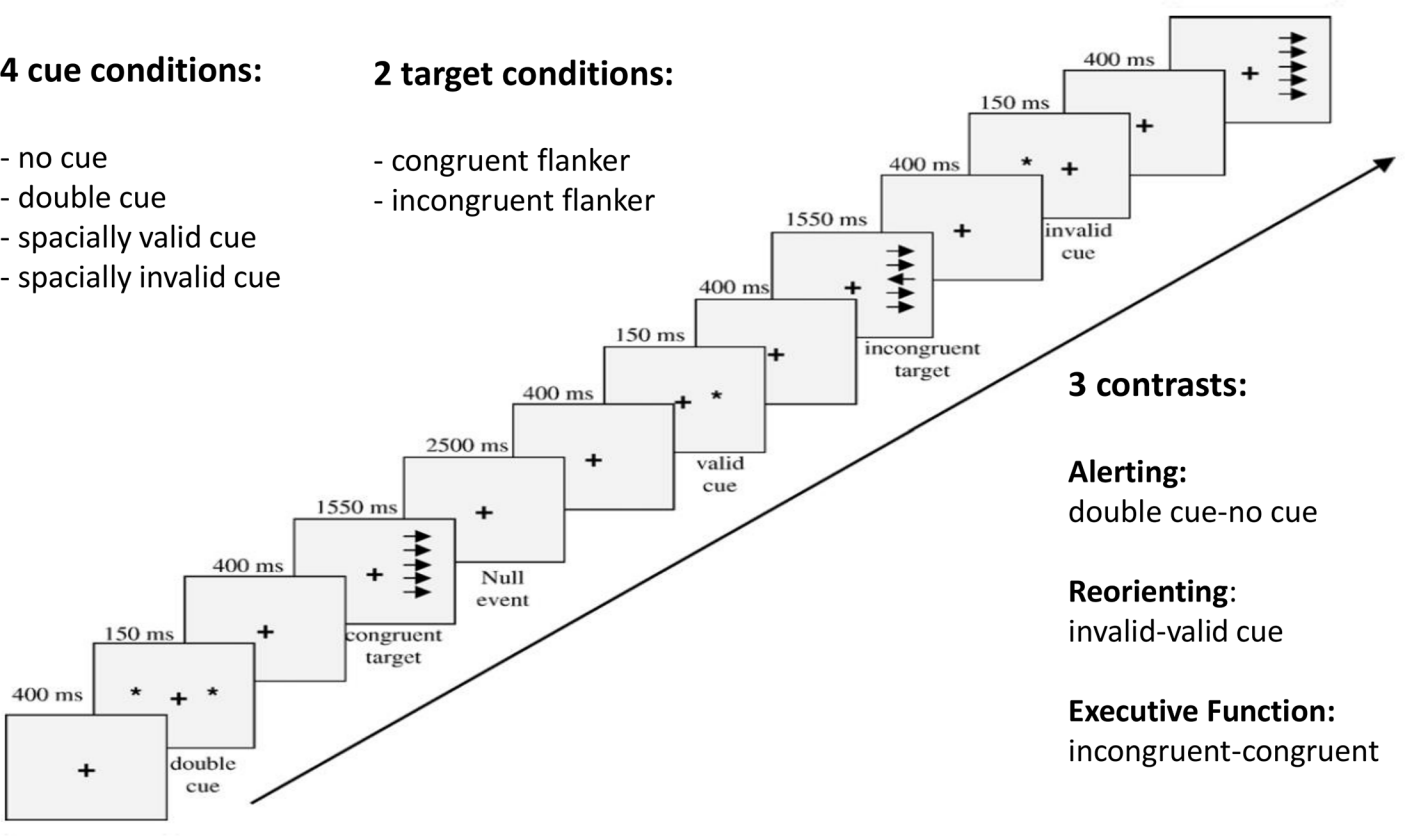
Paradigm of the Attention Network Task. A timeline of the task with several examples is shown from the lower left to the upper right. The task combines 4 cue conditions with 2 target conditions, which enables all 3 contrasts to be tested within a single experiment, thus eliminating the potential variance introduced by combining different tasks.

Stimuli were presented using Presentation software (Neurobehavioral Systems Inc., Albany, USA). We used a modified version of the Attention Network Task (ANT) [24] developed by Fan et al. [20]. We increased the number of trials by 33% to increase statistical power. During this covert attention task, the participants maintain fixation on a centrally located cross and respond as fast as possible via button press to indicate the direction in which the middle arrow (i.e., the target) of five vertically arranged arrows pointed (Fig 1 and S3 Table). Arrows appeared laterally, either on the left or on the right side of the fixation cross, and can be congruent or incongruent with respect to the target. During each trial, the fixation cross was first presented for 400 ms, followed by a 150-ms cue, followed by another 400 ms fixation, and finally target and flanker arrows for a duration of 1550 ms. During the cue period, one of four possible cues was presented: a non-spatially informative double cue, a valid or invalid spatial cue or no cue at all. In total, 224 trials were presented: 112 congruent flankers and 112 incongruent flankers. Twenty-four % of blank trials (i.e. a fixation cross instead of cue and target periods) were included, to avoid habituation effects. The order of trial types was randomized. Prior to scanning, the participants were informed about the different trial types. They were told that spatial cues were highly informative (80% correct) and were encouraged to use these cues to improve performance.

### Behavioral and fMRI analysis

Clinical and behavioral variables for BN and HC groups were compared using two sample Student’s t-tests corrected for 11 multiple comparisons (p<0.004, resulting in a Bonferroni corrected threshold of p<0.05). Behavioral efficiency was assessed by measuring how reaction times (RTs) were influenced by non-spatial warning cues (double versus no cue: Alerting network), spatially valid versus invalid cues (Reorienting network) and congruent versus incongruent flankers (Executive control network) and expressed as subtraction (“network”) scores (for more details, see Fig 1). To consider potential age-related effects we analyzed Pearson correlation coefficients between age and task performance for BN and HC, however, none showed significant effects. We then analyzed correlation coefficients between clinical parameters and task performance (the three attention network scores, mean total RT of all trials and total standard deviations of all trials in the patient group). Clinical parameters were eating-disorder symptoms (Structured Interview for Anorexia and Bulimia Nervosa (SIAB) total score), binging and purging frequencies (SIAB items), ADHD symptoms (Wender Reimherr Interview (WRI) total score), and impulsivity symptoms (Barratt Impulsivity Scale (BIS 10) total score). To control for 25 multiple comparisons of correlation coefficients, p<0.002 was the criterion for significance (resulting in a Bonferroni corrected threshold of p<0.05). Effect sizes of previous behavioral studies, including ours [5,9], were all small to medium, thus we did not necessarily expect significant behavioral group differences related to attentional functions in our fMRI sample (n = 40) (for a power analysis, see the supplemental material). However, several studies investigating brain mechanisms during attention or executive control tasks in similarly sized samples of 16–20 patients in ADHD and BN demonstrated between-group differences at the neural level even if behavioral differences did not reach significance [15,20,24]. Thus, for specific tasks which have been shown previously to uncover deficits in larger samples and which are of high relevance for the onset or maintenance of the disorder, between-group differences at the neural level can also be expected in smaller samples.

Rapid event-related first-level analyses were performed for each participant using Brainvoyager Software (version 2.4, Brain Innovation B.V., Maastricht, the Netherlands [http://www.brainvoyager.com]). The image preprocessing comprised correcting for slice-time effect and movement using rigid-body transformation motion-detection and sinc-transformation motion correction. The images were temporally high-pass filtered at 2 cycles to remove non-linear trends and smoothed with an 8-mm Gaussian kernel and rigid-body transformed to Thalairach space. Predictors were modeled using a general linear model with 8 predictors in a factorial design of four cue conditions (double cue, no cue, spatially valid and spatially invalid cue) and two target conditions (congruent and incongruent flankers) resulting in beta-values of activation for each combination of cue and target condition (4 x 2) at each voxel in the brain. These beta-values thus represent how active each voxel is in a given condition. Correct and incorrect trials were modelled and timed to cue onset. Analysis then proceeded using the correct trials only.

### Hypothesis testing

The alerting network is reflected by the contrast between double cue and no cue events, the reorienting network by the contrast between spatially invalid and spatially valid cue events, and the executive control network is reflected by the contrast between incongruent and congruent target events [19]. Three whole-brain random effects ANOVA were used to determine group differences between the BN and HC groups for all three contrasts, Alerting, Reorienting and Executive Control with the beta values of the general linear model as dependent variable and the relevant conditions (e.g. all “double cue” and all “no cue” for Alerting) and group as independent variables as implemented in the Brainvoyager Software. Group activation contrasts were thresholded at p < 0.01 (voxel level) and corrected for multiple comparisons using minimum cluster thresholds (>40 voxels). The cluster threshold was determined using the Monte Carlo simulation-option of Brainvoyager with 1000 iterations. This approach resulted in an overall chance-level of p<0.05 for each cluster identified. Clusters were then anatomically labeled using the “Thalairach client” software (http://www.talairach.org/client.html). To correct for the potential confounds comorbid depression or anxiety, ANCOVAs for these contrasts was also calculated using BDI-2 scores for depressive symptoms or SCL-90 anxiety scores as covariates, respectively. In addition, three ANOVA contrasts were recalculated excluding BN patients with major depression, ADHD or those on medication.

### Exploratory analyses

In addition, exploratory correlations on brain activations and clinical parameters were calculated within the patient group only. For this purpose, we extracted the beta values by averaging activations of all the significant voxels in the cortical regions of interests (ROIs) that were observed to be different between the BN and HC groups for alerting, reorienting and executive control. We again used SIAB-total score for eating-disorder symptoms, SIAB items for binging and purging, WRI-total score for ADHD symptoms and BIS-10 for impulsivity. To exclude potential confounding effects, we corrected for BDI-2 depression, SCL-90 anxiety, age and BMI scores.

## Results

### Behavioral Results

BN patients showed more eating-disorder symptoms, higher ADHD-scores (particularly inattention) and showed higher impulsivity and more depressive and anxiety symptoms compared with HC even after correcting for multiple comparisons (Table 1).

Both groups performed as expected across the various conditions: responding more quickly to double cues than to no cues (Alerting: double cue < no cue), showing shorter reaction times for valid than invalid targets (Reorienting: valid < invalid cues), and shorter reaction times for congruent compared to incongruent targets (Executive Control: congruent < incongruent targets; for both the BN and HC groups, all p<0.001).

We observed no significant group differences specific to cue or target type for any of the three attention networks based on reaction times, standard deviations of reaction times or errors (see Table 1). In patients with BN, the total mean reaction times (RT-total) and within-subject RT variance (SD-total) correlated with purging frequency (r = 0.765, p<0.001; r = 0.489, p = 0.029, respectively), the Alerting network score correlated with binging frequency and global eating disorder symptoms (SIAB-total; r = 0.518, p = 0.012; r = 0.46, p = 0.048, respectively) and the Reorienting network score correlated inversely with ADHD symptoms (WRI-total; r = -0.491, p = 0.039). However, after correcting for multiple comparisons, only the first correlation remained significant.

### A priori hypotheses

#### Differences in neural attention networks

**Fig 2:**
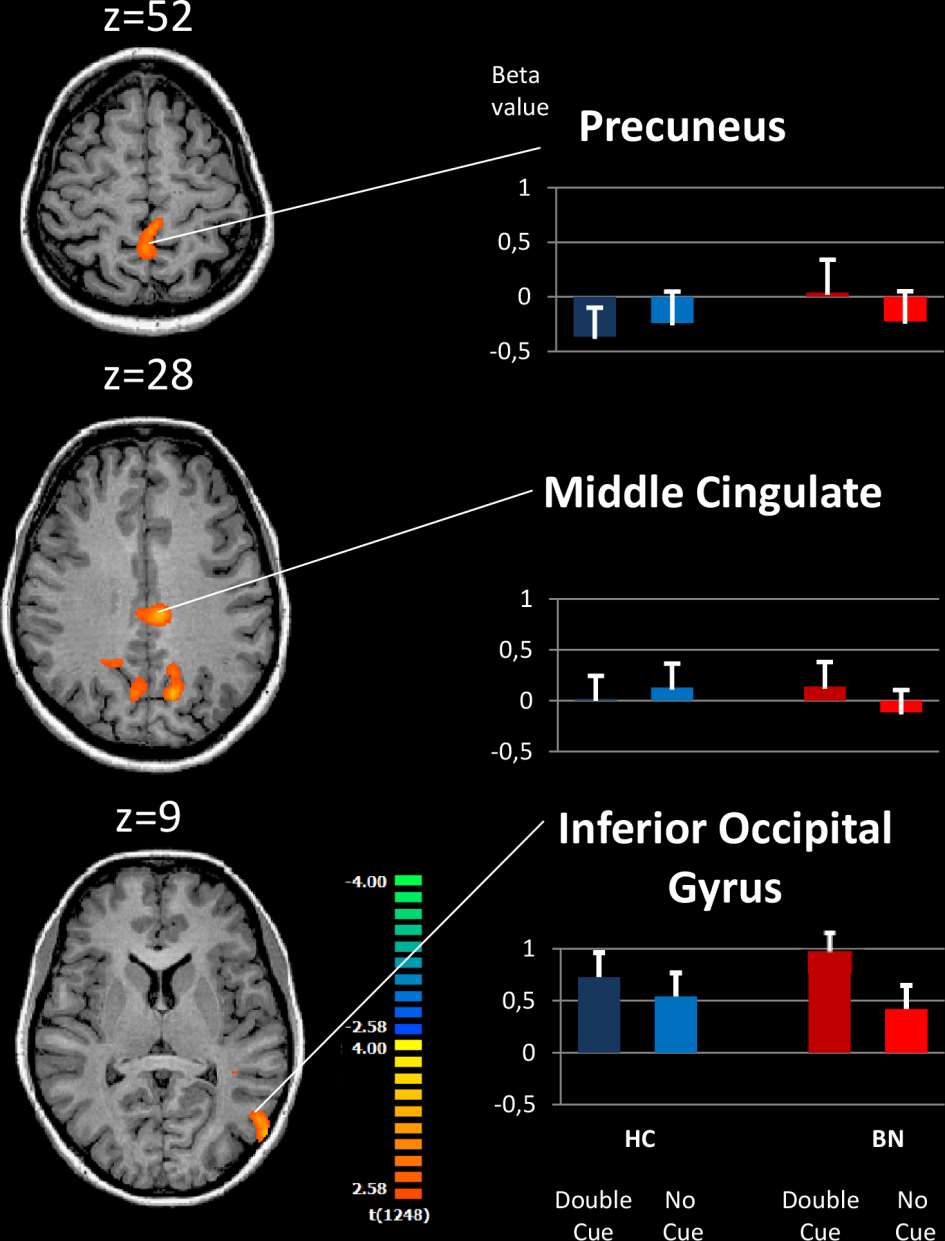
Brain activation during Alertingcontrast for patients with bulimia nervosa relative to healthy controls. The images show three axial slices positioned superiorly to inferiorly from top to bottom. For complete results, please refer to Table 2. Alerting contrast Double Cue–No Cue. The whole-brain analysis was corrected for multiple comparisons using cluster thresholding of 40 voxels. Right, the mean beta values extracted from the regions that differed significantly between the groups. BN: Bulimia nervosa, HC: Healthy controls, ACC: Anterior Cingulate Cortex.

**Fig 3:**
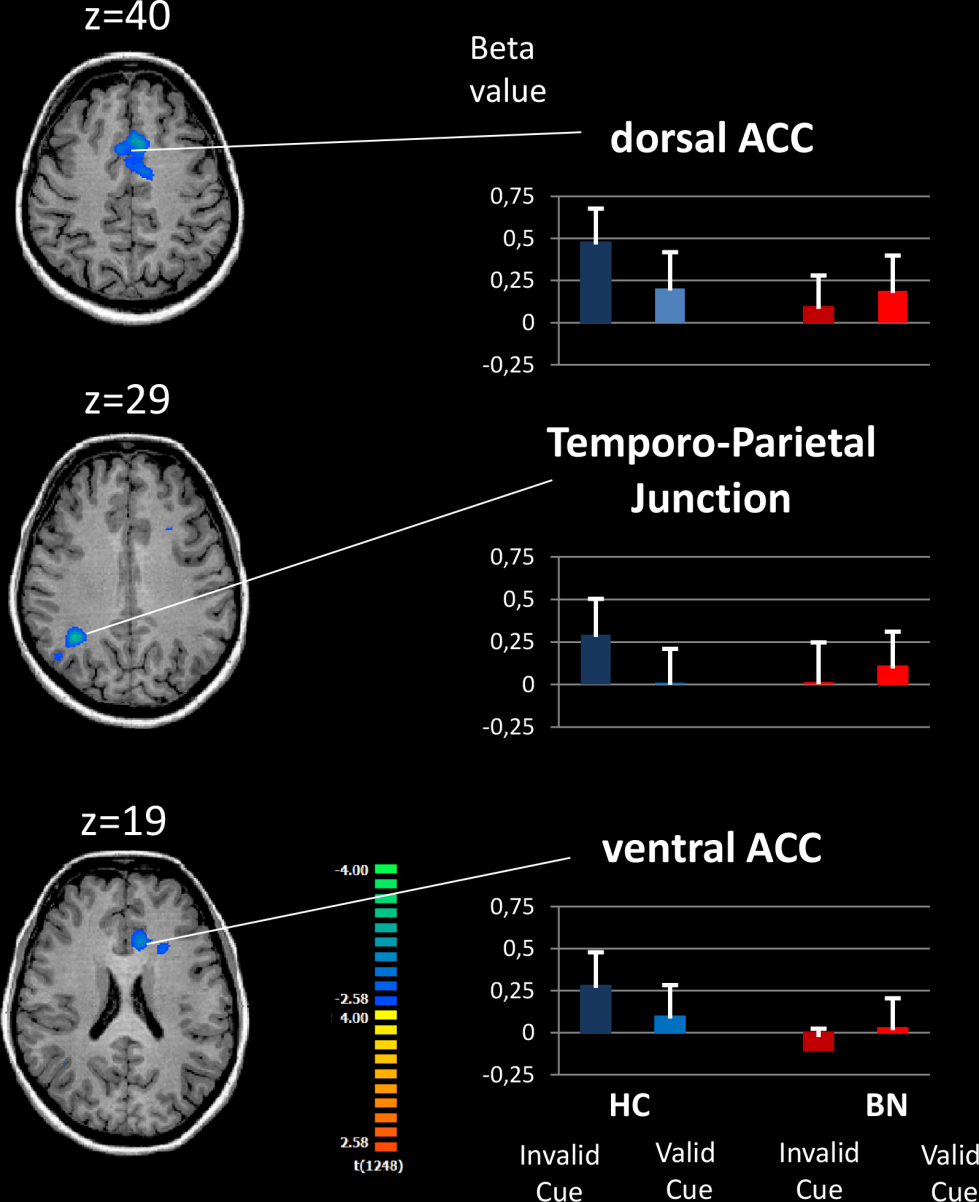
Brain activation during Reorienting contrasts for patient with bulimia nervosa relative to healthy controls. The images show three axial slices positioned superiorly to inferiorly from top to bottom. For complete results, please refer to Table 2. Reorienting contrast Spatially Invalid Cue–Spatially Valid cue. The whole-brain analysis was corrected for multiple comparisons using cluster thresholding of 40 voxels. Right, the mean beta values extracted from the regions that differed significantly between the groups. BN: Bulimia nervosa, HC: Healthy controls, ACC: Anterior Cingulate Cortex.

**Fig 4:**
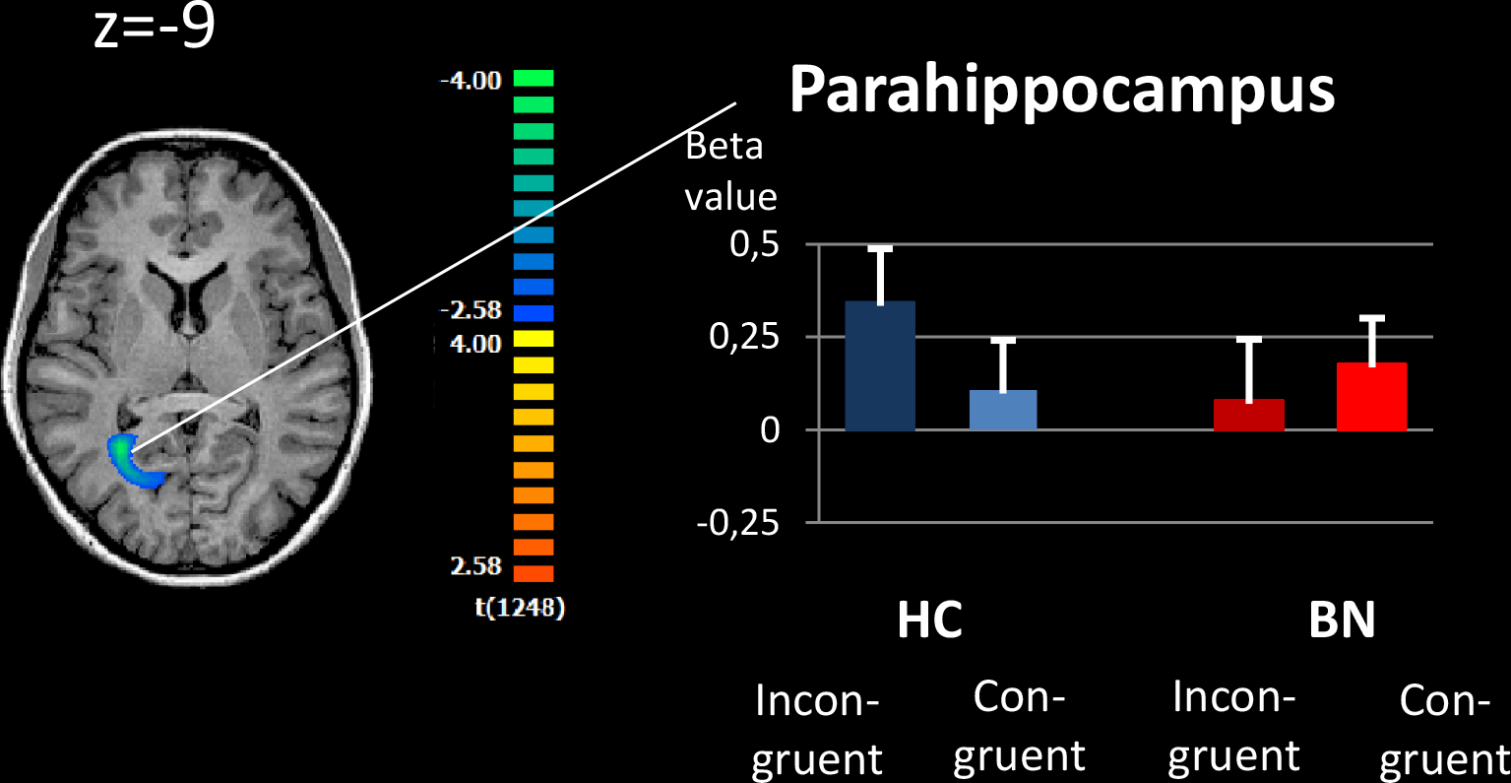
Brain activation during Executive Control contrast for patients with bulimia nervosa relative to healthy controls. The image shows an axial slices. For complete results, please refer to Table 2. Executive control contrast Incongruent Target–Congruent Target. The whole-brain analysis was corrected for multiple comparisons using cluster thresholding of 40 voxels. Right, the mean beta values extracted from the region that differed significantly between the groups. BN: Bulimia nervosa, HC: Healthy controls, ACC: Anterior Cingulate Cortex.

We observed significant group differences in neural activations in all three attention networks (Figs 2–4 and Table 2).

**Table 2:** Significant activation differences between BN and HC.

Area	Side	x	y	z	Brodman Area	Cluster size	Mean t	Mean p
**A) Alerting Network**								
Posterior Insula	Left	-31	-41	18	BA 13	1742	3.124	0.005
Inferior Occipital Gyrus	Left	-55	-68	12	BA 39	1161	2.957	0.006
Middle Cingulate Gyrus	Left (bil)	-7	-23	27	BA 23	1200	2.885	0.007
Paracentral Lobule	Left	-7	-32	57	BA 6	2391	2.881	0.007
Precuneus	Left	-13	-65	30	BA 7	1355	2.857	0.008
Cuneus	Right	8	-65	30	BA 7	1982	2.758	0.009
**B) Reorienting Network**								
Temporo-Parietal Junction	Right	35	-47	30	BA 40	1080	-2.883	0.008
Dorsal Anterior Cingulate	Left (bil)	-3	10	40	BA 32	3303	-2.832	0.008
Cerebellar Tonsil	Right	26	-56	-42		1613	-2.822	0.008
Cerebellar Culmen	Left	-78				1376	-2.788	0.009
Ventral Anterior Cingulate	Left	-14	23	22	BA 32	1223	-2.772	0.009
**C) Executive Control**								
Parahippocampal Gyrus	Right	29	-50	9	BA 30	2035	-2.984	0.006

All regions of interest are listed with the name of their respective Talairach coordinates, Brodman area, cluster size, average t-Values and p-values. Bil.: bilateral.

#### Alerting

(Fig 2) With respect to the alerting network (double cue–no cue), the BN participants showed increased neural activity in a parietal and occipital brain regions compared with the HCs. These areas included brain regions that have been associated with alerting functions in previous studies, such as the inferior occipital cortex [20]. In addition, hyperactivation was noted in occipital and parietal areas, such as the inferior occipital gyrus, middle cingulate and para-central gyrus. In addition, less deactivation was observed in patients with BN compared with HCs in the left and right precuneus, an area typically part of the default mode network (DMN) activation and being deactivated during active tasks. The inverse group comparison showed no significant difference (HC > BN).

#### Reorienting

(Fig 3) With respect to the reorienting network (invalid cue–valid cue), the between-group comparisons revealed reduced neural activation in BN participants compared with HCs in areas including the right temporo-parietal junction (TPJ), the cerebellum and the anterior cingulate cortex (ACC). The inverse group comparison showed no significant difference (HC > BN).

#### Executive Control

(Fig 4) With regard to the executive control network (incongruent–congruent target conditions), we observed reduced neural activation in patients with BN compared with HCs in the right parahippocampal gyrus. Again, we did not find any significant differences in the inverse comparison (HC > BN).

#### Medication and comorbidity effects

(S1–S5 Figs) A comparison of activation maps corrected for depression and anxiety symptoms resulted in similar activation patterns for all of the attention networks albeit with smaller cluster sizes in some cases. The only exception was the Alerting contrast corrected for anxiety: The initial threshold had to be lowered to p<0.05 to reveal similar activation patterns. Additionally, a reanalysis excluding participants with major depression, ADHD or on SSRI medication also showed similar results, again with smaller cluster sizes in some cases.

### Exploratory correlation analyses

**Fig 5:**
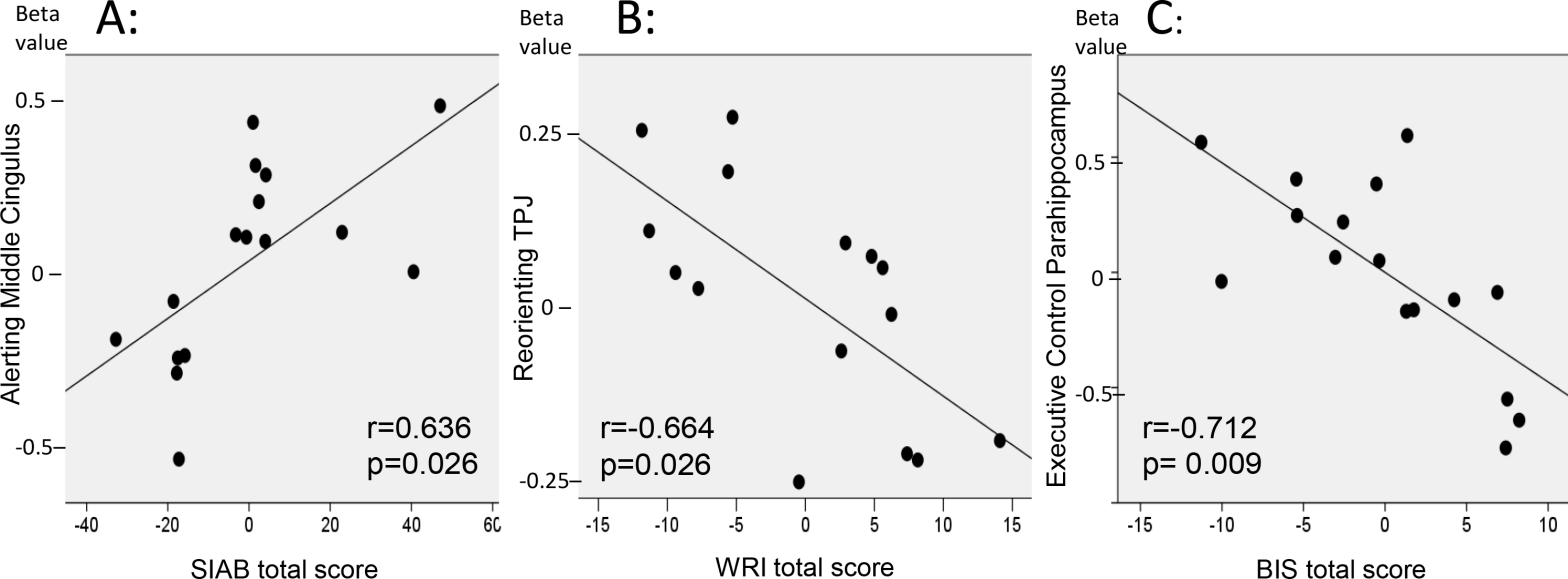
Analysis of correlation with clinical parameters. Brain activations extracted from areas significantly different between BN and HC for Alerting, Reorienting and Executive Control were correlated with clinical parameters. Correlations were corrected for age, BMI, depression and anxiety scores. A: Hyperactive during Alerting, bilateral posterior cingulate correlated with global eating-disorder symptoms (SIAB total score); B: Hypoactive during Reorienting, temporo Parietal Junction (TPJ) correlated inversely with ADHD scores (WRI-total); C: Hypoactive during Executive Control, parahippocampal gyrus correlated inversely with impulsivity scores (BIS-total).

We observed significant associations between neural activity during Alerting in the bilateral middle cingulate and the global eating disorder symptoms, as measured by the SIAB total score (r = 0.636, p = 0.026, see Fig 5A). With respect to Reorienting, neural activity in right TPJ was inversely correlated with ADHD-symptoms, as measured by the WRI-total score (r = -0.664, p = 0.026, Fig 5B). With regard to Executive Control, neural activity in right parahippocampus was inversely correlated with BIS-impulsivity scores (r = -0.712, p = 0.009, Fig 5C).

## Discussion

To our knowledge, this study is the first thorough investigation of attention brain networks in patients with BN using fMRI. During the Attention Network Task, BN and HC groups showed significant differences in their neural activation patterns, but not in behavioral task performance. In both groups, alerting, reorienting and executive behavioral network scores (i.e. RT difference scores of the respective conditions) were significantly above zero, indicating that task manipulations were successful across both groups. Overall longer RTs and larger within-subject RT variance correlated with more purging in the preceding two weeks before measurement in the patient group. In addition, an increased Alerting effect correlated with binging and overall eating pathology, albeit only without correcting for multiple comparisons.

The BN patients showed hyperactivation in a broad network associated with alerting accompanied by reduced deactivation in typical DMN areas. Hypoactivation was noted in the reorienting network in the ACC, temporo-parietal junction (TPJ) and parahippocampal areas and in the parahippocampus during executive control.

Our findings suggest that patients with BN are characterized by dysfunctional neural activation patterns that underlie different attention networks and that are also associated with the severity of eating pathology, ADHD-symptoms and impulsivity. More specifically, it appears that during alerting, some brain areas related to the DMN network are not deactivated to a similar extent as in controls leading to broad hyperactivation of the parieto-occipital cortex. By contrast, during reorienting of attention, key brain regions, such as the TPJ were hypoactived in patients with BN compared to controls. Together, this pattern may reflect an ongoing internal preoccupation, e.g. with food and body-image-related thoughts, with more effort required to shift away from them and attend to the task at hand [27]. Our results regarding the executive control network replicate and extend previous findings of hypoactivity in associated brain circuits which has been linked to impaired inhibition, binge-type eating and other impulsive behaviors in BN individuals [15,16]. For the first time, we could demonstrate that dysfunctional attentional network functioning in BN is not limited to the executive attentional domain but rather extends to alerting and reorienting attentional systems.

### Alerting

The alerting network was shown to be hyperactive in BN patients. This hyperactivation was noted in regions typically active during Alerting, such as the parietal cortex [20], but also in other regions, such as the occipital cortex, the posterior cingulate gyrus and posterior insula. Interestingly, eating-disorder severity correlated with both inefficient processing of stimuli (as evidenced by longer RTs and larger SDs) but also increased Alerting effects (also described as phasic alertness [28]). Notably, larger Alerting effects as found in patients with BN are associated with better performance for cued trials. A possible explanation may be the increased levels of anxiety observed in the BN patient group, which is consistent with recent findings, showing that anxiety may lead to increased autonomic arousal and improved efficiency of alerting network functions reflecting a possible hypervigilant state [29]. Indeed, when correcting for anxiety-symptoms, hyperactivation during Alerting was found to be less prominent, pointing to an additional partial role of anxiety in neural (hyper-) activation (S2 Fig). Patients with more severe eating-disorder symptoms showed more neural hyperactivation of the middle cingulate. In addition to hyperactivation in such typical areas of the alertness network, further compensatory areas (i.e. in the occipital cortex) were recruited in BN individuals. In support of that notion, increased levels of neural activation in the occipital cortex of BN individuals have been described previously by Uher et al. [30]: BN patients were shown pictures of food and aversive emotional images and were reported to have greater occipital activation upon viewing pictures of food. Our results extend these findings beyond food stimuli and point to an even more basic level of impairment in BN individuals. Similarly, hyperactivation of the visual cortex indicating possible compensatory mechanisms of disturbed attention networks has been reported in adult patients with ADHD during inhibition [31], working memory and attentional tasks, [32] (for a review see [33]).

Furthermore, unlike HCs, BN patients did not demonstrate task-related deactivation of the precuneus during alerting. The precuneus is part of the DMN and has been associated with self-referential processing, being particularly active during periods of rest and exhibiting decreases in activity during goal-directed actions [34]. Thus, it appears that BN patients do not switch their neural resources as effectively to task-specific networks.One possible explanation may be that patients with BN report being constantly preoccupied with self-related food- and weight-concerns [1]. Such internal and self-referential thoughts, in particular self-ascriptions, first-person perspective and self-conscious awareness typically result in activation of the precuneus [35,36]. In concert with the observed hyperactivation in the alerting network, it might be speculated that constant ongoing pervasive thoughts result in a failure to properly deactivate the DMN during attention tasks and create a need for increased effort to maintain alertness via hyperactivation of the alerting network. In support of this interpretation, neural hyperactivity during Alerting, longer RTs, and larger within-subject RT variances were associated with more severe eating-disorder symptoms. Findings of reduced DMN deactivation are consistent with findings of previous studies. For example, Marsh et al. [15] reported decreased DMN deactivations in BN individuals also during executive control tasks. Ongoing activity in the DMN and missing neural resources may also interfere and exacerbate hypoactivation in higher-order cortical networks [36]. The deactivation of the DMN and its inverse correlation with task-positive networks was significantly correlated with task performance in several studies [37]. Thus, Sonuga-Barke et al. formulated a default-mode interference hypothesis for ADHD [38]. Diminished DMN suppression has been noted in ADHD patients and has been observed to correlate with attentional lapses [36].

### Reorienting and Executive Control

The reorienting and executive control networks were observed to be hypoactive in BN patients, as compared with the HC. The differences were noted in areas typically involved in reorienting and executive control, such as the TPJ and the ACC [20]. The right TPJ is a key area for reorienting attention [20,32] and is important for the development of joint attention and theory-of-mind processes [39,40]. BN patients have shown impaired emotional awareness of themselves and others [41] and show impaired recognition of facial emotions [42]; however, these findings were mixed (for a recent review see [43]). McAdams et al. noted the TPJ to be significantly less active in an fMRI social recognition task [44] suggesting altered neural correlates of theory-of-mind in BN individuals. These hypoactivations observed in our experiment suggest fundamentally impaired attention processes that might underlie the development of higher-order social attention deficits in BN individuals.

The parahippocampal region, which is hypoactive in BN individuals during executive control tasks, is close to the hippocampal region identified by Mash et al. [15] during the Simon Spatial Task and may similarly correlate with the patients’ difficulties with impulsivity and decision making, as suspected in their study [4,9]. Support for this hypothesis comes from our finding that reduced executive control network activation was indeed correlated with BIS-impulsivity scores in our study. The evidence of hypoactivation in cingular regions is consistent with prior findings in BN individuals [15,16] and has been linked to insufficient self-regulatory control, which promotes impulsive behavior including the binge eating and emotional dysregulation often observed in BN individuals.

Our results are also consistent with recent morphological findings reported by Marsh et al. [27] in patients with BN when compared with HC. They showed changes in surface morphology with volume reduction in the inferior frontal, temporo-parietal and cingulate cortex whereas the volume of the occipital cortex was increased. In their study, volume loss in the inferior frontal cortex positively correlated with illness duration and symptom severity and with functional results from a Stroop interference test. The pattern of their morphological findings fits well with our functional pattern with respect to hypoactivation in our study being associated with volume reduction in Marsh et al.’s study and hyperactivation being linked to volume increase. Thus, the altered basic neural attentional processes in our study may play a role in the cause and may also be a result of the morphological differences uncovered when patients with BN are compared with HC. Previous studies with ADHD patients also showed hypoactivation in fronto-cingular brain areas paralleled by hyperactivation in occipital areas. This pattern of neural dysfunction was linked to inattention and impulsivity (for a recent meta-analysis see [45]), traits shared by patients with BN.

### Implications and future directions

The strength of this study is that a detailed assessment of attentional functions grounded on a neuroscientifically based model of attention was combined with fMRI and a thorough clinical assessment of the BN patients and controls. This design enabled an investigation of the potential connection between the altered neural patterns of attentional dysfunction and relevant clinical symptoms within the same sample. Our results clearly demonstrate dysfunction in brain networks of attention in female patients with BN, even in the absence of behavioral task differences in our sample. No inferences can be made for male patients. We also replicated the finding of increased clinical symptoms of ADHD in this patient group. The failure to properly deactivate the DMN and the increased neural resources required to reach alertness in patients with BN may be manifestations of their difficulty to shift attention away from BN-related thoughts. Hypoactivation of fronto-parietal networks including ACC, TPJ and parahippocampus during reorienting and executive control tasks may underlie the inattentive symptoms, impulsivity and emotion-regulation problems in this group. These results add to the growing literature implicating attention deficits in BN [4–7] and have important implications for researchers and clinicians alike. Researchers investigating neural correlates of causes and consequences of BN should include differential alterations of attention networks into pathophysiological models of BN. Future studies should add additional clinical control groups which was unfortunately beyond the scope of the current study. In particular, clinical groups with ADHD, but without eating disorder, as well as BN groups both with and without comorbid ADHD should be directly compared. This comparison would allow to answer the question whether overall shared deficits can be observed in attention networks, or whether ADHD- and BN-specific dysfunctions can be clearly separated.

Clinicians should carefully diagnose inattention and comorbid ADHD in patients with BN. These patients show higher symptom severity of eating disorders and have a generally higher psychopathological burden [3,5]; they may also benefit from additional ADHD interventions. For example, attention deficits may be addressed in specific cognitive remediation therapies for BN [46]. Several case series also suggest a potential role for stimulants (e.g. [47–49]) and show a marked reduction in ADHD symptoms and in binging and purging while the patients maintained a stable weight. However, systematic randomized intervention studies are required to further validate these findings.

## Supporting Information

S1 Fig Brain activation corrected for depressive symptoms. This figure shows the areas that differ significantly between the BN and HC groups for the Alerting, Reorienting and Executive Control contrast when controlling for depressive symptoms. The images show axial slices positioned superiorly to inferiorly from top to bottom. The whole-brain analysis was corrected for multiple comparisons using a more lenient cluster thresholding of 10 voxels. BN: Bulimia nervosa, HC: Healthy controls, ACC: Anterior Cingulate Cortex.
(TIF)

S2 Fig Brain activation corrected for anxious symptoms. This figure shows the areas that differ significantly between the BN and HC groups for the Alerting, Reorienting and Executive Control contrast when controlling for anxious symptoms. The images show axial slices positioned superiorly to inferiorly from top to bottom. The whole-brain analysis was corrected for multiple comparisons using a more lenient cluster thresholding of 10 voxels. BN: Bulimia nervosa, HC: Healthy controls, ACC: Anterior Cingulate Cortex.
(TIF)

S3 Fig Group differences in brain activation when patients with major depression are excluded. This figure shows the areas that differ significantly between the BN and HC groups for the Alerting, Reorienting and Executive Control contrast when 4 patients with major depression are excluded. The images show axial slices positioned superiorly to inferiorly from top to bottom. The whole-brain analysis was corrected for multiple comparisons using a more lenient cluster thresholding of 10 voxels. BN: Bulimia nervosa, HC: Healthy controls, ACC: Anterior Cingulate Cortex.
(TIF)

S4 Fig Group differences in brain activation when patients with ADHD are excluded. This figure shows the areas that differ significantly between the BN and HC groups for the Alerting, Reorienting and Executive Control contrast when 2 patients with ADHD are excluded. The images show axial slices positioned superiorly to inferiorly from top to bottom. The whole-brain analysis was corrected for multiple comparisons using a more lenient cluster thresholding of 10 voxels. BN: Bulimia nervosa, HC: Healthy controls, ACC: Anterior Cingulate Cortex.
(TIF)

S5 Fig Group differences in brain activation when patients taking medication are excluded. This figure shows the areas that differ significantly between the BN and HC groups for the Alerting, Reorienting and Executive Control contrast when 5 patients taking serotonin reuptake inhibitors are excluded. The images show axial slices positioned superiorly to inferiorly from top to bottom. The whole-brain analysis was corrected for multiple comparisons using a more lenient cluster thresholding of 10 voxels. BN: Bulimia nervosa, HC: Healthy controls, ACC: Anterior Cingulate Cortex.
(TIF)

S1 File Additional information on recruiting, stimuli, data acquisition and processing. (DOCX)

S1 Table Clinical variables without patients with major depression. SD: Standard deviation; T: Student’s t-test T-Value; df: degrees of freedom; p: p-value, uncorrected; WRI: Wender-Reimherr Interview for ADHD; BIS: Barratt Impulsivity Scale; BDI: Beck’s Depression Inventory; SCL-90: Symptoms Check List.
(DOCX)

S2 Table Clinical variables without without patients with ADHD. SD: Standard deviation; T: Student’s t-test T-Value; df: degrees of freedom; p: p-value, uncorrected; WRI: Wender-Reimherr Interview for ADHD; BIS: Barratt Impulsivity Scale; BDI: Beck’s Depression Inventory; SCL-90: Symptoms Check List.
(DOCX)

S3 Table List of Stimuli. Note that half of the stimuli were presented on either side.
(DOCX)
