# Supplementary figures and images for: Attention Network Dysfunction in Bulimia Nervosa - An fMRI Study
Source: PLoS One. 2016 Sep 8;11(9):e0161329. doi: 10.1371/journal.pone.0161329 (PMC5015972; doi:10.1371/journal.pone.0161329)

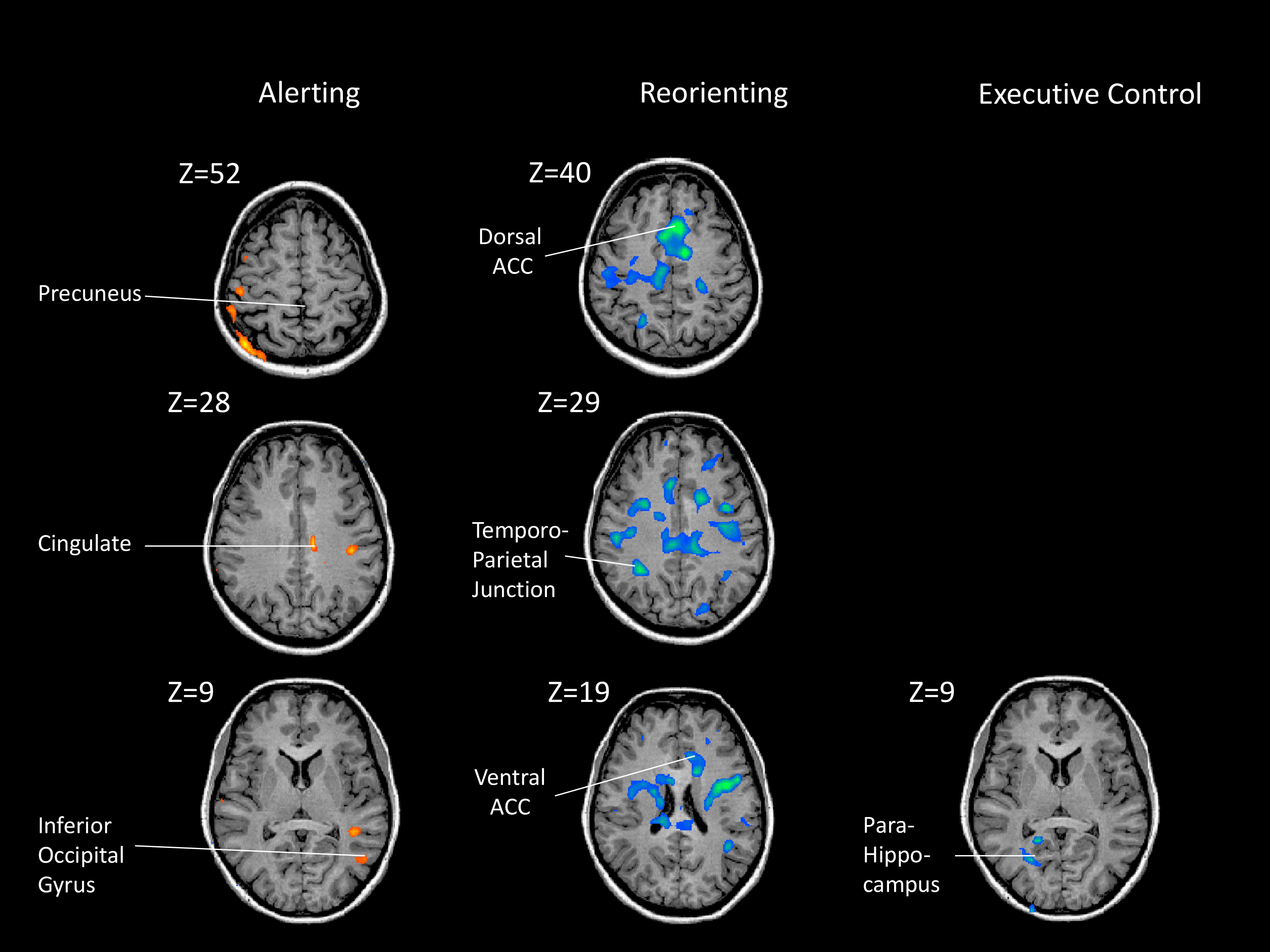

Supplement: S1 Fig — This figure shows the areas that differ significantly between the BN and HC groups for the Alerting, Reorienting and Executive Control contrast when controlling for depressive symptoms. The images show axial slices positioned superiorly to inferiorly from top to bottom. The whole-brain analysis was corrected for multiple comparisons using a more lenient cluster thresholding of 10 voxels. BN: Bulimia nervosa, HC: Healthy controls, ACC: Anterior Cingulate Cortex. (TIF) [file pone.0161329.s001.tif]

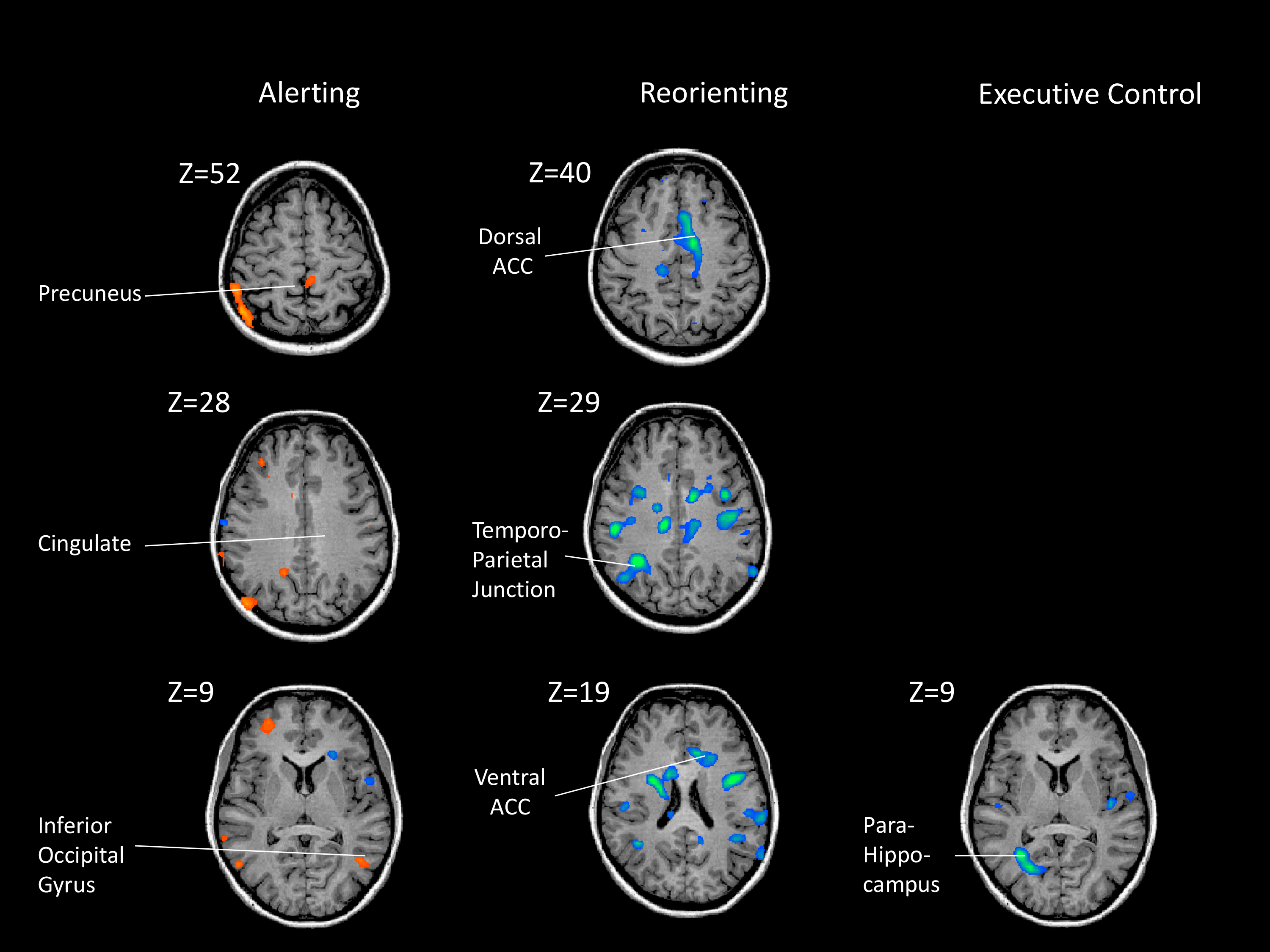

Supplement: S2 Fig — This figure shows the areas that differ significantly between the BN and HC groups for the Alerting, Reorienting and Executive Control contrast when controlling for anxious symptoms. The images show axial slices positioned superiorly to inferiorly from top to bottom. The whole-brain analysis was corrected for multiple comparisons using a more lenient cluster thresholding of 10 voxels. BN: Bulimia nervosa, HC: Healthy controls, ACC: Anterior Cingulate Cortex. (TIF) [file pone.0161329.s002.tif]

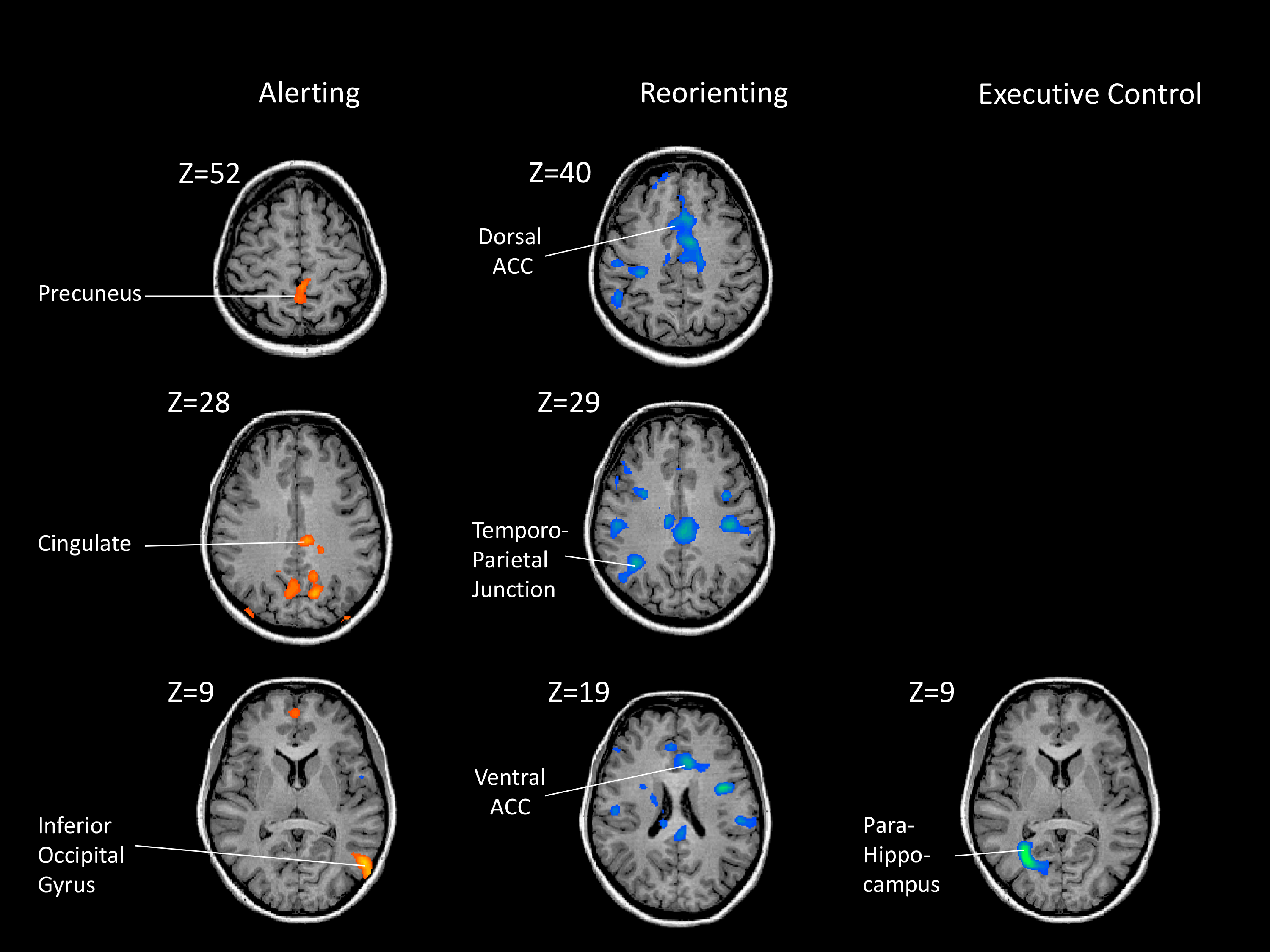

Supplement: S3 Fig — This figure shows the areas that differ significantly between the BN and HC groups for the Alerting, Reorienting and Executive Control contrast when 4 patients with major depression are excluded. The images show axial slices positioned superiorly to inferiorly from top to bottom. The whole-brain analysis was corrected for multiple comparisons using a more lenient cluster thresholding of 10 voxels. BN: Bulimia nervosa, HC: Healthy controls, ACC: Anterior Cingulate Cortex. (TIF) [file pone.0161329.s003.tif]

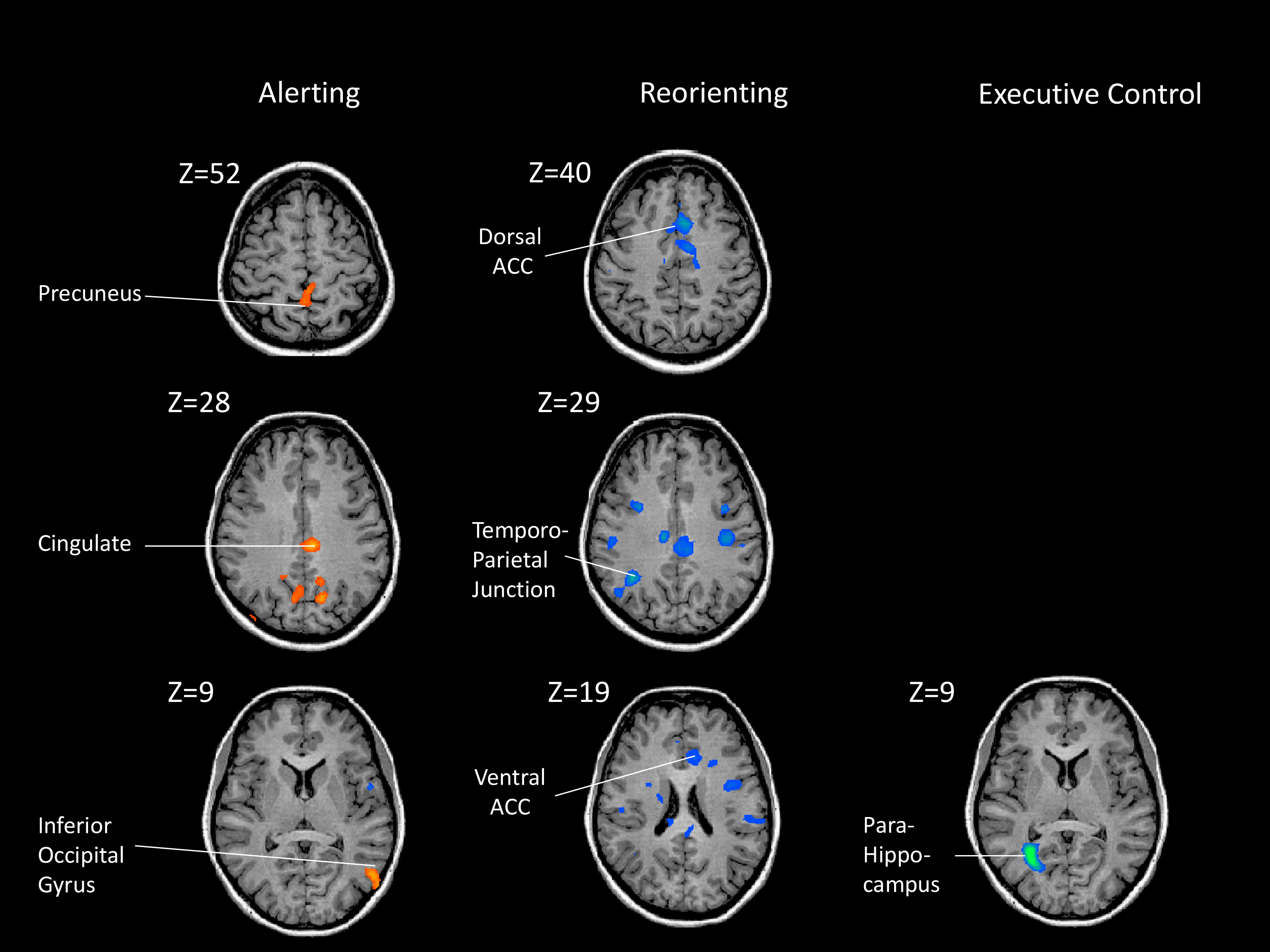

Supplement: S4 Fig — This figure shows the areas that differ significantly between the BN and HC groups for the Alerting, Reorienting and Executive Control contrast when 2 patients with ADHD are excluded. The images show axial slices positioned superiorly to inferiorly from top to bottom. The whole-brain analysis was corrected for multiple comparisons using a more lenient cluster thresholding of 10 voxels. BN: Bulimia nervosa, HC: Healthy controls, ACC: Anterior Cingulate Cortex. (TIF) [file pone.0161329.s004.tif]

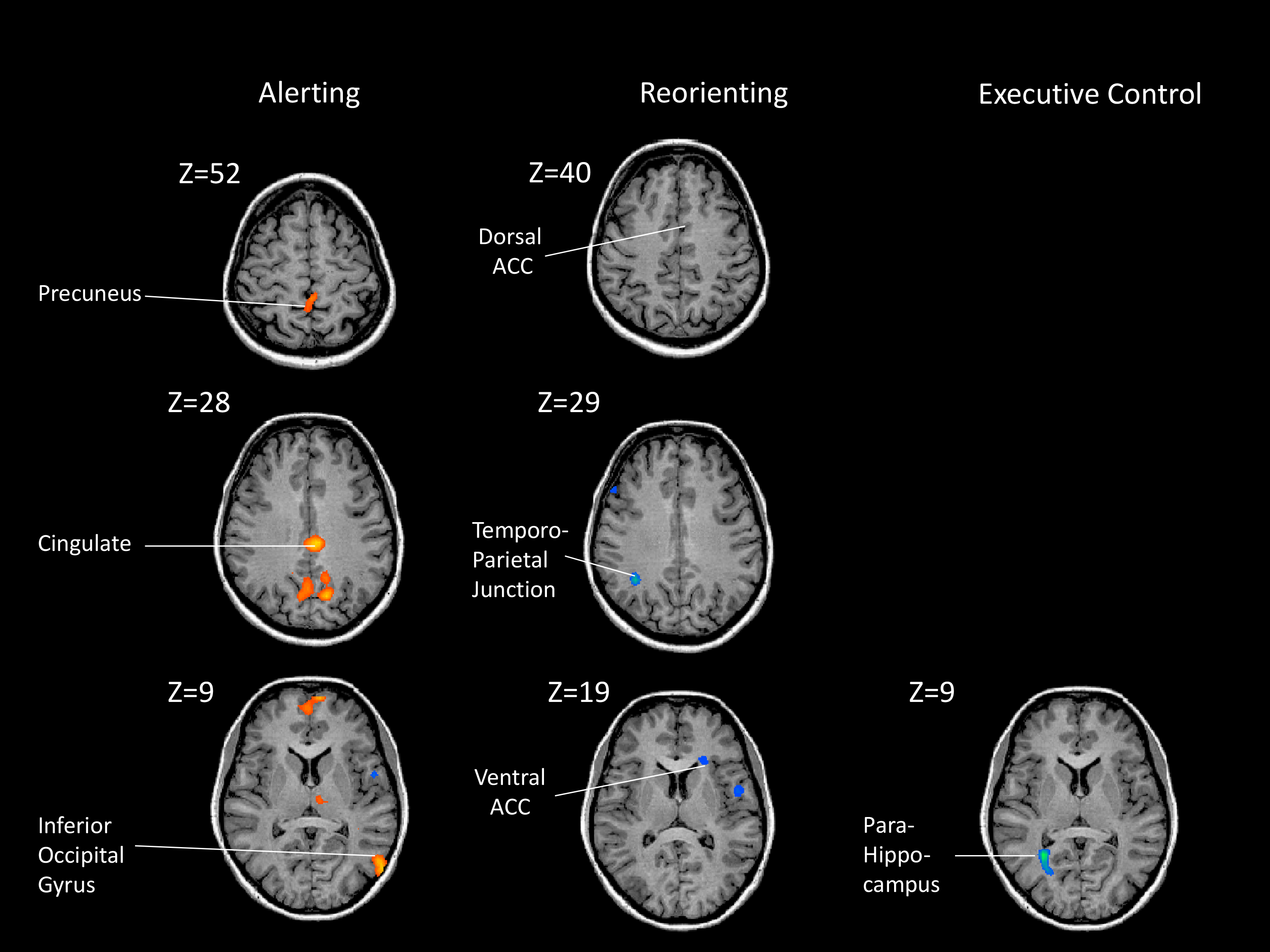

Supplement: S5 Fig — This figure shows the areas that differ significantly between the BN and HC groups for the Alerting, Reorienting and Executive Control contrast when 5 patients taking serotonin reuptake inhibitors are excluded. The images show axial slices positioned superiorly to inferiorly from top to bottom. The whole-brain analysis was corrected for multiple comparisons using a more lenient cluster thresholding of 10 voxels. BN: Bulimia nervosa, HC: Healthy controls, ACC: Anterior Cingulate Cortex. (TIF) [file pone.0161329.s005.tif]
