# Supplementary material for: Attention Network Dysfunction in Bulimia Nervosa - An fMRI Study
Source: PLoS One. 2016 Sep 8;11(9):e0161329. doi: 10.1371/journal.pone.0161329 (PMC5015972; doi:10.1371/journal.pone.0161329)
Supplement: S1 File — (DOCX) [file pone.0161329.s006.docx]

**Supplementary information for Seitz et al., Attention Network Dysfunction in Bulimia Nervosa - An fMRI study**

**Supplemental Methods**

Participants

The exclusion criteria for the patients were a history of psychosis, substance abuse and IQ<80. The exclusion criteria for the healthy controls were any psychiatric diagnoses and IQ<80. Bulimia Nervosa was formally diagnosed using the Structured Clinical Interview for DSM-IV Disorders (SKID). The severity of the eating disorder symptoms in the participants was assessed using the Eating Disorder Inventory-II (EDI-II) and the Structured Interview for Anorexia and Bulimia (SIAB-Ex). The Beck Depression Inventory-II (BDI-II) and the Symptom-Checklist (SCL-90) were used to document depressive and anxious symptomatology. The Barratt Impulsivity Scale-10 (BIS-10) was used to assess impulsiveness. Attention-deficit and Hyperactivity Disorder symptomatology was diagnosed in three ways: childhood ADHD was assessed using the Wender-Utah Revised Checklist for Childhood (WURS-K), and current ADHD symptoms were assessed using the ADHD-SB self-rating scale and the Wender-Reimherr Interview (WRI). IQ was measured using the Mehrfachwahl-Wortschatz-Intelligenztest (MWT-B) (Lehrl, 2005) or the Hamburg-Wechsler Inelligenztest for children (HAWIK).

Stimuli

The stimuli were projected onto a set of goggles worn by each of the participants in the MRI scanner. The responses in the scanner were collected via two button-presses with the right hand using a keyboard that rested at the subject’s side. Each stimulus comprised a vertical row of five black visually presented arrows, which pointed to the left or to the right against a white background. The target was the middle arrow, which was flanked by two arrows above and two arrows below that pointed in the same direction (congruent condition) or in the opposite direction (incongruent condition). The stimuli (one central arrow plus four flankers) comprised a total visual angle of 4.34° vertically and 14.5° horizontally. Each participant was required to indicate the direction of the middle arrow by pressing the left key for the target pointing to the left or the other key for the target pointing to the right.

Image Acquisition and Preprocessing

The scanning was performed using a 3.0 Tesla Siemens Trio Tim Scanner at Aachen University Hospital. Anatomical scans were acquired over 176 slices using an MP-Rage T1-weighted sequence with a time of repetition (TR) of 1.88 sec at a 1x1x1-mm resolution in a 256x256 matrix. Functional scans covering the entire brain used T2*-weighted echo-planar imaging (EPI) with TE=28 ms, TR=2000, matrix: 64x64, 34 slices, flip angle=77 degrees at an oblique orientation to minimize frontal artifacts, which resulted in a voxel size of 3x3x3.6 mm³ and a field of view (FOV) of 192 mm. In total, 395 EPI volumes were acquired for each subject.

The image preprocessing comprised correcting for slice-time effect and movement using rigid-body transformation motion-detection and sinc-transformation motion correction (version 2.4, Brain Innovation B.V., Maastricht [[http://www.brainvoyager.com](http://www.brainvoyager.com/)]). The time-series data at each voxel were preprocessed to correct for slice-time effect and movement and were temporally filtered and smoothed using an 8-mm FWHM Gaussian kernel. The functional data were then coregistered with the anatomical volume and transferred into standard stereotaxic space using Talairach normalization (Talairach and Tournoux, 1988). Statistical maps were generated using a general linear model with 8 predictors differentiating 4 cue conditions (double cue, no cue, spatially valid and spatially invalid cue) and two target conditions (congruent and incongruent flankers). All of these events were then convolved using the canonical hemodynamic response function. Correct and incorrect trials were modeled, and only correct trials were used for further analyses.

Behavioral Analyses

The efficiency of the various attentional networks was assessed behaviorally by measuring how reaction times (RTs) were influenced by non-spatial warning cues (double cue), spatially valid and invalid cues, and congruent versus incongruent flankers. The following cognitive scores were calculated to deduce the behavioral efficiency of the three postulated attentional networks (Posner & Petersen, 1990): the alerting effect was calculated by subtracting the mean RT of the double cue trials from the mean RT of the no-cue trials over all of the congruent/incongruent flanker trials; the reorienting effect was considered to be the difference between the mean RT of invalidly cued trials and the mean RT of validly cued trials over all of the congruent/incongruent flanker trials; the executive control (conflict) effect was measured as the difference between the mean RT of all incongruent flanker trials and the mean RT of all congruent flanker trials, across all cue types.

MRI analysis

To exclude influences of comorbid depression or medication, we conducted several post hoc analyses to confirm that our findings on the a priori hypothesis were robust and not dependent on comorbidities or medication effects. First, we calculated whole-brain ANCOVAs for all three contrasts, Alerting, Reorienting and Executive Control, and compared the BN group with the HC group, entering the BDI depression and SCL-90 anxiety scores, separately, as covariates. Second, we excluded the patients with a diagnosis of major depression and those taking medication and recalculated our original ANOVA for all three contrasts to compare the BN and HC groups. Analyses are presented with the same initial threshold of p<0.01 and then corrected with a more lenient cluster threshold of 10 instead of 40 voxels to enable the recognition of similar activation patterns as in the main analysis (except anxiety-corrected ANCOVA Alerting contrast, where p<0.05 was used as the initial threshold).

**Supplemental Results**

BDI- and SCL-90 Anxiety-corrected ANCOVAs and the ANOVA that excluded depressed and medicated patients with BN revealed similar brain activations to those observed in the primary analysis (see Supplementary Figures 1-5).
