# Supplementary material for: Attention Network Dysfunction in Bulimia Nervosa - An fMRI Study
Source: PLoS One. 2016 Sep 8;11(9):e0161329. doi: 10.1371/journal.pone.0161329 (PMC5015972; doi:10.1371/journal.pone.0161329)
Supplement: S1 Table — SD: Standard deviation; T: Student’s t-test T-Value; df: degrees of freedom; p: p-value, uncorrected; WRI: Wender-Reimherr Interview for ADHD; BIS: Barratt Impulsivity Scale; BDI: Beck’s Depression Inventory; SCL-90: Symptoms Check List. (DOCX) [file pone.0161329.s007.docx]

|  | Bulimia Nervosa (N=16) | | Healthy controls (N=20) | | Analysis | | |
| --- | --- | --- | --- | --- | --- | --- | --- |
|  | Mean | SD | Mean | SD | t | df | p |
| WRI total score | 20.80 | 13.655 | 6.40 | 4.68 | -4.40 | 32 | 0.000 |
| BIS impulsivity score | 61.78 | 12.01 | 51.15 | 9.61 | 2.86 | 32 | 0.007 |
| BDI-2 depression score | 19.33 | 13.44 | 3.95 | 5.22 | 4.68 | 32 | 0.000 |
| SCL-90 anxiety score | 6.14 | 9.14 | 1.45 | 2.01 | 2.32 | 32 | 0.033 |

**Supplementary Tables**

**S1 Table:** Clinical variables without patients with major depression
